# Supplementary figures and images for: A Msp1-containing complex removes orphaned proteins in the mitochondrial outer membrane of T. brucei
Source: Life Sci Alliance. 2023 Aug 16;6(11):e202302004. doi: 10.26508/lsa.202302004 (PMC10432679; doi:10.26508/lsa.202302004)

kDa

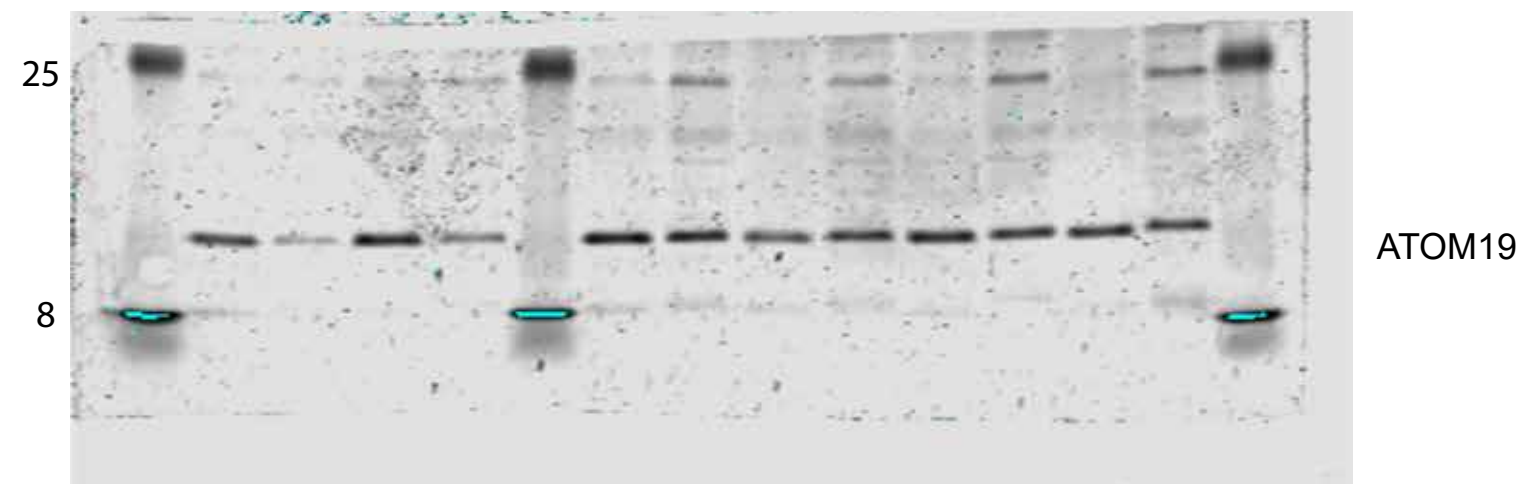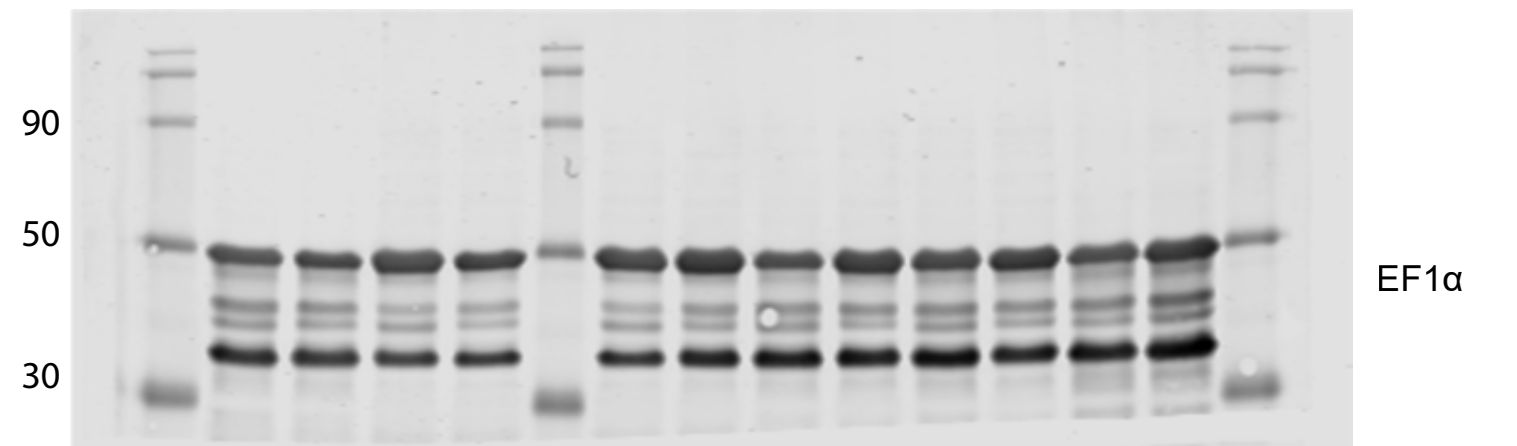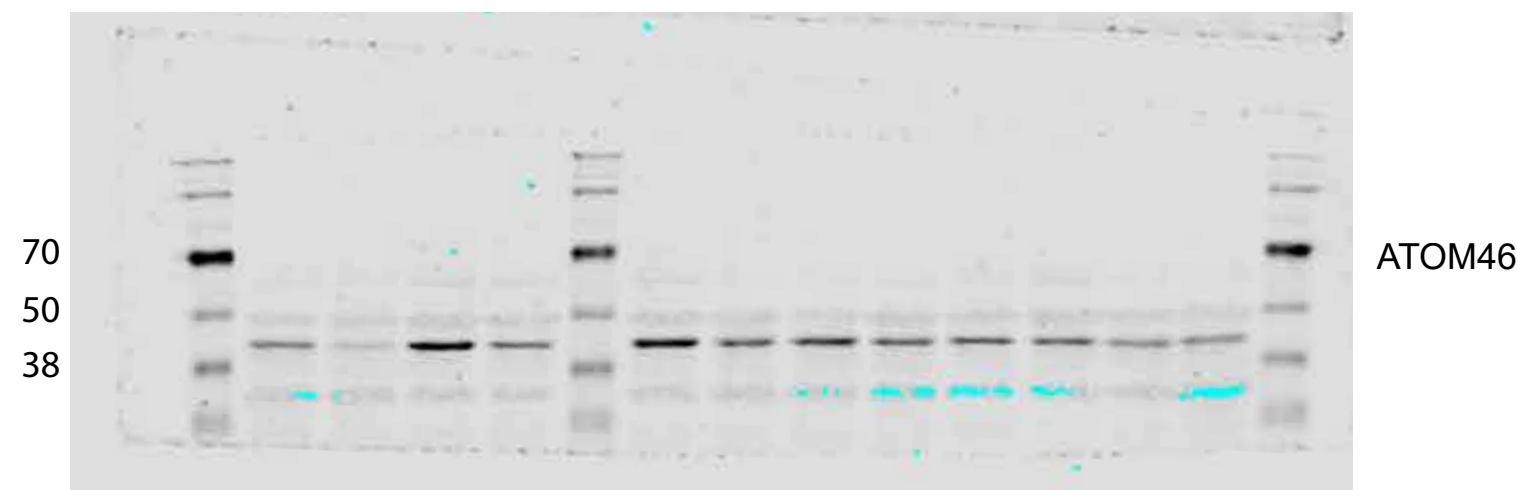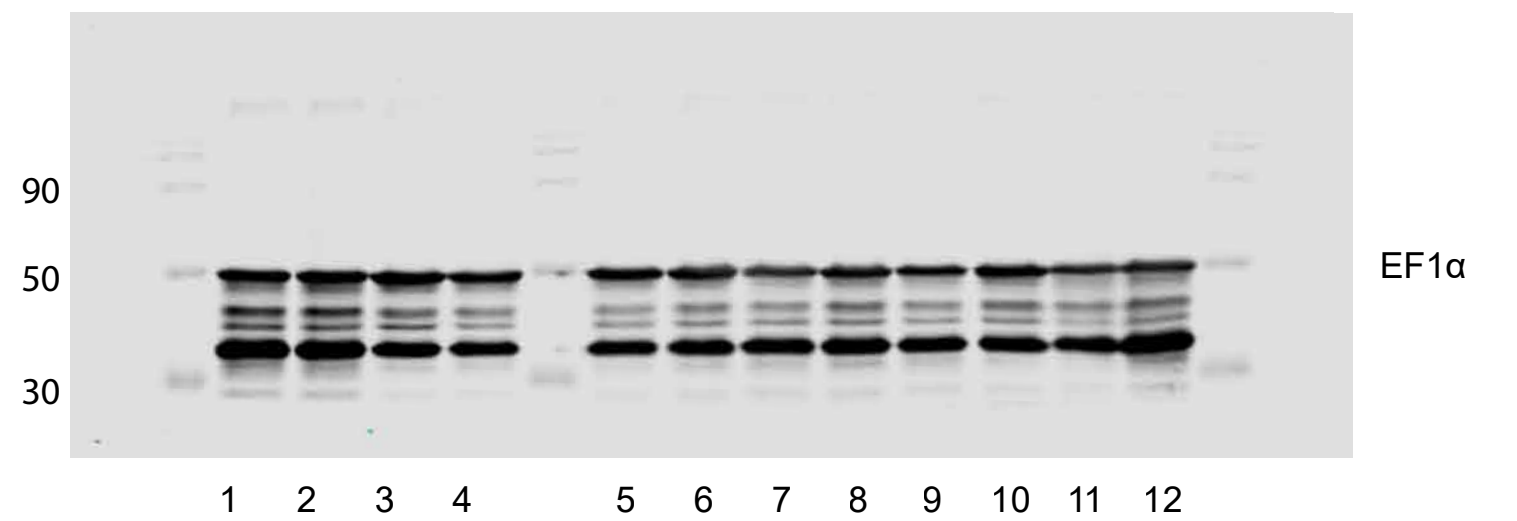

Supplement: Supplementary file 1 [file LSA-2023-02004_SdataF5.pdf]
